# Efficacy of acupuncture combined with traditional Chinese herbal for primary epilepsy patients with cognitive impairment: A protocol for systematic review and meta-analysis

**DOI:** 10.1371/journal.pone.0297410

**Published:** 2024-07-01

**Authors:** Hua Xue, Li Zeng, Hongxian He, Dongxun Xu

**Affiliations:** 1 Department of Neurology, Sichuan Taikang Hospital, Chengdu, Sichuan, China; 2 Department of Rehabilitation, Affiliated Hospital of Yunnan University, Kunming, Yunnan, China; UCSI University, MALAYSIA

## Abstract

**Background:**

Epilepsy is a common and serious chronic neurological disorder, and some patients suffer from cognitive dysfunction. We aim to assess the efficacy and safety of acupuncture combined with traditional Chinese herbal for primary epilepsy patients with cognitive impairment.

**Methods:**

To search the randomized control trials (RCTs) published before April 20, 2023 from PubMed, Embase, Cochrane Library, Chinese Biomedical Literature Database (CBM), China National Knowledge Infrastructure (CNKI), Web of science, and Wanfang Database. The risk of bias within each individual trial was evaluated using the Cochrane Collaboration tool. RevMan5.3 software was used for statistical analysis. The odds ratio (OR) or weighted mean difference (WMD) with a 95% confidence interval (CI) was calculated for each RCT before data pooling.

**Results:**

The primary outcomes involve changes in cognitive function and behavioral disturbances. The secondary outcomes focused on quality of life and adverse effects.

**Conclusion:**

The results of this review are expected to provide new guidelines for the treatment of primary epilepsy patients with cognitive impairment.

**Trial registration:**

This systematic review protocol was registered at the International Prospective Register of Systematic Reviews (PROSPERO) (Registration number: CRD42023415355).

## 1. Introduction

Epilepsy is a chronic disease in which sudden abnormal discharge of brain neurons leads to transient brain dysfunction [1]. Each epileptic seizure of the patient is unpredictable, which not only seriously reduces the quality of life, but also causes different degrees of cognitive dysfunction, manifested as a functional decline in attention, memory, abstract thinking ability, reasoning ability, etc. even lost [2, 3]. Epidemiological studies revealed that the global prevalence of epilepsy has surpassed 70 million, with approximately 700,000 new cases reported annually [2]. Numerous experimental models have demonstrated that cognitive dysfunction is a common comorbidity in epilepsy. For instance, Kumar et al. discovered that all rats with pentetrazole-induced epilepsy exhibited cognitive deficits [4]. In clinical practice, the prevalence of cognitive impairment in epilepsy patients varies from 30% to 40%, depending on the assessment method and population under evaluation [5]. Cognitive impairment significantly impacts the quality of life for individuals with epilepsy, emphasizing the significance of early prediction and intervention in managing cognitive dysfunction. With the promotion and application of the “biology-psychology-social” medical model, in the process of clinical diagnosis and treatment of epilepsy, it is necessary to pay attention not only to the control of epileptic seizures but also to the accompanying cognitive impairment of patients.

The earliest records of epilepsy in China can be traced back to 2000 years ago in the Huang Di Nai Jing of traditional Chinese medicine. Traditional Chinese medicine has a long history and rich experience in the treatment of epilepsy. It has the advantages of a stable curative effect, few side effects, and can promote the recovery of brain cell function, and is expected to be applied in antiepileptic therapy [6, 7]. Acupuncture is another important traditional healing method, which has been used and applied for more than 2000 years in China. In recent years, acupuncture has shown positive therapeutic effects in improving overall effectiveness of epilepsy treatment, reducing the severity of epileptic seizures, and minimizing adverse reactions. Research has confirmed that electroacupuncture stimulation of “Zu-san-li” and “Shang-ju-xu” for 6 weeks significantly reduces the level of cyclooxygenase-2 (COX-2) in the hippocampus of epileptic rats, resulting in an anti-inflammatory effect [8]. Furthermore, electroacupuncture intervention has been found to upregulate the expression of Nuclear factor erythroid2-related factor 2 (Nrf-2) and its downstream antioxidant factors, activating the Nrf-2-ARE signaling pathway to protect brain tissue against oxidative stress damage [9]. In summary, acupuncture’ s mechanism in treating epilepsy encompasses various aspects of the condition’ s pathogenesis, including apoptosis inhibition, inflammatory response suppression, oxidative stress reduction, ion channel regulation, and modulation of intestinal flora. However, high-quality evidence from relevant studies is lacking. Therefore, we carried out a meta-analysis of randomized controlled trials (RCTs) to reach a solid conclusion with a larger sample size. We aimed to determine the effects of acupuncture for epilepsy patients with cognitive impairment and provide evidence-based recommendations for patients with epilepsy.

## 2. Methods

### 2.1 Study registration

This systematic review protocol was registered at PROSPERO (Registration number: CRD42023415355). The protocol is according to Preferred Reporting Items for Systematic Reviews and Meta-Analysis (PRISMA) statement guidelines. The results of this meta-analysis will be published in a journal or conferences.

### 2.2 Inclusion criteria

#### 2.2.1 Participants

Patients who are clinically diagnosed with epilepsy patients with cognitive impairment according to the diagnostic criteria of the 10TH revision of the International Classification of Diseases (ICD-10) (World Health Organization, 1992) and the Diagnostic and Statistical Manual of Mental Disorders (DSM-IV) (American Psychiatric Association, 1994) will be included. The onset of cognitive impairment was required to be definitely and causally linked with primary epilepsy. Additionally, patients with combined heart, lung, kidney, liver, bone marrow, blood, and other systemic diseases, combined intracranial occupying lesions, combined cerebral infarction, combined hysteria, schizophrenia, and another psychiatric history should be excluded.

#### 2.2.2 Interventions

For the experimental group, trials that use acupuncture therapy combined with traditional Chinese herbs will be included; and acupuncture therapy involves manual acupuncture, electroacupuncture, fire acupuncture, warm acupuncture, and scalp acupuncture. For the corresponding control group, interventions could be placebo or waiting list control, sham acupuncture, conventional treatment, or pharmacotherapy that consists of the experimental group.

#### 2.2.3 Outcome indicators

The primary outcomes focused on cognitive function and behavioral disturbances, which could be measured by scales such as the EpiTrack score, the Mini-mental state examination (MMSE), the Hasegawa’s Dementia Scale (HDS), the Montreal Cognitive Assessment (MoCA) score. The secondary outcomes focused on quality of life and adverse effects which could be measured by scales such as the Quality of Life Scale for Epileptic Patients-31 (QOLIE-31), the short form 36 health survey questionnaire (SF-36), the Activities of Daily Living (ADL) Scale, and the Functional Activities Questionnaire (FAQ).

#### 2.2.4 Study type

Randomized controlled trials (RCTs) related to epilepsy patients with cognitive impairment will be included without language restrictions. Non-RCTs, inappropriate intervention, uncontrolled trials, reviews, and protocols for RCTs were excluded.

### 2.3 Data sources and search strategies

All published RCTs in the following databases will be searched from their inception to April 2023: PubMed, EMBASE, Web of Science, Chinese Biomedical Literature Database, the Cochrane Central Register of Controlled Trials (CENTRAL), Wanfang database, and the China National Knowledge Infrastructure (CNKI), without language restrictions.

The search terms included “epilepsy”, “Epilepsies”, “Seizure Disorder”, “Seizure Disorders”, “Cognitive Dysfunctions”, “Cognitive Impairments”, “Cognitive Impairment”, “Cognitive Disorder”, “Cognitive Decline”, “Cognitive Declines”, “Mental Deterioration”, “Acupuncture Therapy”, “Acupuncture”, “Traditional Chinese medicine”, “Traditional Chinese herbs”. An example of the search strategy for PubMed is summarized in Table 1.

**Table 1:** Search strategy for PubMed.

Number	Search terms (PubMed)
1	Epilepsy[Mesh]
2	Epilepsy[Title/Abstract]
3	Seizure Disorder [Title/Abstract]
4	Seizure Disorders [Title/Abstract]
5	Seizure [Title/Abstract]
6	1 or 2–5
7	Cognitive Dysfunctions [Mesh]
8	Cognitive Dysfunctions [Title/Abstract]
9	Cognitive Impairments [Title/Abstract]
10	Cognitive Impairment [Title/Abstract]
11	Cognitive Disorder [Title/Abstract]
12	Cognitive Decline [Title/Abstract]
13	Cognitive Declines [Title/Abstract]
14	Mental Deterioration [Title/Abstract]
15	7 or 8–14
16	Acupuncture [Mesh]
17	Acupuncture Therapy [Title/Abstract]
18	Acupuncture Treatment [Title/Abstract]
19	Acupuncture Therapy [Title/Abstract]
20	Pharmacoacupuncture Treatment [Title/Abstract]
22	Acupotomy [Title/Abstract]
22	Fire acupuncture [Title/Abstract]
23	Warm acupuncture [Title/Abstract]
24	Scalp acupuncture [Title/Abstract]
25	Acupuncture [Title/Abstract]
26	16 or 17–25
27	Traditional Chinese Medicine [Mesh]
28	Traditional Chinese Medicine [Title/Abstract]
29	Chinese Herbal Drugs [Title/Abstract]
30	Chinese Herbal [Title/Abstract]
31	27 or 28–30
32	6 and 15 and 26 and 31

### 2.4 Study selection and data extraction

**Fig 1:**
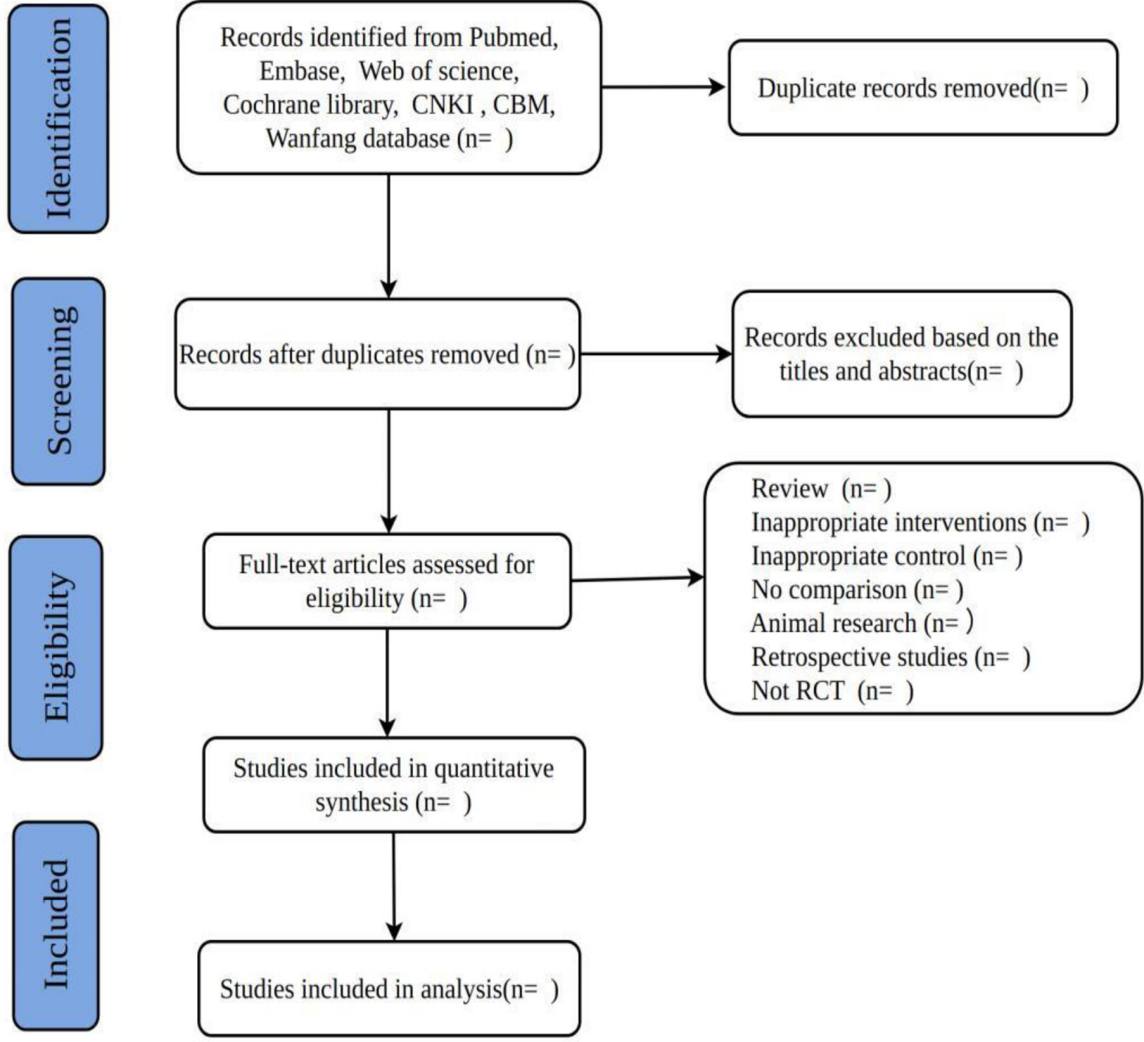
Flowchart of trial selection process for this systematic review. “n” represents the number of studies.

We will import all identified literature into EndNote X9 software to delete any duplicates. Two authors will screen the titles/abstracts of all potential studies to remove studies that are not related to the topic. Then, the full text of the remaining studies will be read carefully to further determine whether they fulfill all eligible criteria. If necessary, a third author will help to solve any divergence between the two authors. The following data were extracted from the included studies: (1) basic information of the study including the first author’s name, year of publication, sample sizes, participant’s age, and disease duration; (2) interventions details; (3) control details; (4) primary outcome indicators, secondary outcome indicators before and after the intervention; (5) adverse events. If available data could not be obtained directly from the article, the corresponding author was contacted and clarification of ambiguities and missing information was provided by phone or email. Details of the selection process will be presented in the PRISMA flow chart (Fig 1).

### 2.5 Methodological quality assessment

The methodological quality of the included references was assessed using the Cochrane Collaboration’s Risk of Bias (ROB) tool by two reviewers independently. This assessment tool mainly including the generation of random sequences, allocation concealment, blinding of researchers and subjects, blinding in outcome assessment, and completeness of outcome data, selective reporting of research results and other sources of bias. Each item is judged as: “low risk”, “high risk” or “unclear”. Any discrepancies concerning the assessments were resolved through the discussion with a methodological researcher.

### 2.6 Statistical analysis

To conduct statistical analysis, we used Review Manager (RevMan) software version 5.3.0 (Cochrane Central Executive Team, United Kingdom) as recommended by Cochrane Collaboration. Heterogeneity was measured using both Cochran’s Q-test (P value ≤0.10 was used to define a significant degree of heterogeneity) and the *I*^*2*^ statistic (*I*^*2*^ >50% showed the existence of heterogeneity). If the heterogeneity was significant (P < 0.1, *I*^*2*^ > 50%), a random effect (RE) model was chosen to pool the data, and if there was acceptable heterogeneity (P ≥ 0.1, *I*^*2*^ ≤ 50%), a fixed effect (FE) model was used. The mean difference (MD) or standard mean difference (SMD) was used to represent continuous data, and the Odds Ratio (OR) were used to represent dichotomous data. Results were reported with 95% confidence intervals (CI), and P < 0.05 was considered a significant statistical effect. Subgroup analyses will be performed for heterogeneity by intervention, type of control (placebo, sham acupuncture, usual care, or drug treatment), duration of treatment, point, and outcome measure. Sensitivity analyses were performed on the primary endpoint to test the homogeneity of the results. After excluding low-quality studies, we will rerun the meta-analysis and use other statistical methods.

### 2.7 Assessment of publication bias

When more than 10 studies were included, publication bias was assessed using funnel plots. We analyzed potential publication bias using Egger or Begg tests and estimated results according to the Cochrane Handbook of Systematic Reviews of Interventions.

### 2.8 Ethics and dissemination

No human or animal subjects or samples were used. Patients did not participate in the formulation of research questions, measurement of outcomes, and study design.

## 3. Discussion

At present, the influencing factors of cognitive dysfunction in epilepsy patients have been basically clarified, but the pathogenesis has not yet been fully clarified. Studies have found that epilepsy-related cognitive impairment may be related to damage such as hippocampal sclerosis, abnormal transmitter transmission, inflammatory factors and oxidative stress [10–14]. D’Avila et al. discovered that activated microglia release various inflammatory mediators, which can lead to tissue damage and neurotoxicity through mechanisms like oxidative stress and synaptic remodeling [15]. These processes have been linked to cognitive impairment. Mishra et al. conducted a study using a mouse model of epilepsy and found that reducing lipid peroxidation levels and increasing antioxidant mechanisms resulted in shorter seizure duration and improved cognitive function [16]. The existence of cognitive impairment will bring a certain burden to the life of epilepsy patients. Therefore, it is of great significance to diagnose and treat cognitive impairment in time to reduce the burden on patients and their families.

Modern pharmacological studies have shown that some traditional Chinese medicines (such as Gastrodia elata, Polygala tenuifolia, Acorus tatarinowii, Nardostachys chinensis, Safflower, etc.) have the effects of inhibiting platelet aggregation, prolonging thrombin time, protecting neurons, nourishing blood and calming the nerves, antiepileptic, sedative, antispasmodic, and analgesic [17–20]. Acupuncture and moxibustion treat epilepsy mainly by disrupting brain waves, inhibiting seizures, affecting the neuroendocrine immune system, and protecting neurons, thereby reducing seizures and improving the quality of life of patients with epilepsy. Acupuncture acts on acupoints through physical stimulation, stimulates related nerve veins, affects the secretion and release of neurotransmitters in the brain, and regulates the electrical activity of brain nerve cells [9, 21].

However, there is no studies have not been systematically organized on the efficacy of acupuncture for primary epilepsy patients with cognitive impairment. Thus, it is necessary to organize evidence that has been proven by RCTs, so that it can be used.

## Supporting information

S1 Checklist PRISMA 2009 checklist. (DOC)
